# The commensal bacterium *Lactiplantibacillus plantarum* imprints innate memory-like responses in mononuclear phagocytes

**DOI:** 10.1080/19490976.2021.1939598

**Published:** 2021-07-05

**Authors:** Aize Pellon, Diego Barriales, Ainize Peña-Cearra, Janire Castelo-Careaga, Ainhoa Palacios, Nerea Lopez, Estibaliz Atondo, Miguel Angel Pascual-Itoiz, Itziar Martín-Ruiz, Leticia Sampedro, Monika Gonzalez-Lopez, Laura Bárcena, Teresa Martín-Mateos, Jose María Landete, Rafael Prados-Rosales, Laura Plaza-Vinuesa, Rosario Muñoz, Blanca de las Rivas, Juan Miguel Rodríguez, Edurne Berra, Ana M. Aransay, Leticia Abecia, Jose Luis Lavín, Hector Rodríguez, Juan Anguita

**Affiliations:** a Inflammation and Macrophage Plasticity Laboratory, CIC bioGUNE-BRTA (Basque Research and Technology Alliance), Derio, Spain; b Faculty of Medicine and Nursing, Universidad Del Pais Vasco (UPV/EHU), Leioa, Spain; c Genomic Analysis Platform, CIC bioGUNE-BRTA, Derio, Spain; d Physiopathology of the Hypoxia-signaling Pathway Laboratory, CIC bioGUNE-BRTA, Derio, Spain; e Departamento De Tecnología De Alimentos, Instituto Nacional De Investigación Y Tecnología Agraria Y Alimentaria (INIA), Madrid, Spain; f Laboratorio De Biotecnología Bacteriana, Instituto De Ciencia Y Tecnología De Alimentos Y Nutrición (ICTAN-CSIC), Madrid, Spain; g Department of Nutrition and Food Science, Universidad Complutense De Madrid, Madrid, Spain; h CIBERehd, ISCIII, Madrid, Spain; i Bioinformatics Unit, CIC bioGUNE-BRTA, Derio, Spain; j Ikerbasque, Basque Foundation for Science, Bilbao, Bizkaia, Spain; k Centre for Host-Microbiome Interactions, Faculty of Dentistry, Oral and Craniofacial Sciences, King’s College London, United Kingdom; RPR: Department of Preventive Medicine and Public Health and Microbiology, Universidad Autónoma De Madrid, Madrid 28029, Spain; JLL: Applied Mathematics Department, Bioinformatics Unit, NEIKER-BRTA, Parque Tecnológico De Bizkaia, Derio, Spain

**Keywords:** *Lactiplantibacillus plantarum*, *Lactobacillus*, monocytes, macrophages, innate immune memory, trained immunity, microbiota, immunometabolism

## Abstract

Gut microbiota is a constant source of antigens and stimuli to which the resident immune system has developed tolerance. However, the mechanisms by which mononuclear phagocytes, specifically monocytes/macrophages, cope with these usually pro-inflammatory signals are poorly understood. Here, we show that innate immune memory promotes anti-inflammatory homeostasis, using as model strains of the commensal bacterium *Lactiplantibacillus plantarum*. Priming of monocytes/macrophages with bacteria, especially in its live form, enhances bacterial intracellular survival and decreases the release of pro-inflammatory signals to the environment, with lower production of TNF and higher levels of IL-10. Analysis of the transcriptomic landscape of these cells shows downregulation of pathways associated with the production of reactive oxygen species (ROS) and the release of cytokines, chemokines and antimicrobial peptides. Indeed, the induction of ROS prevents memory-induced bacterial survival. In addition, there is a dysregulation in gene expression of several metabolic pathways leading to decreased glycolytic and respiratory rates in memory cells. These data support commensal microbe-specific metabolic changes in innate immune memory cells that might contribute to homeostasis in the gut.

## Introduction

The emergence of microbiota research has expanded our knowledge on the role of commensal microorganisms in controlling a wide variety of physiological functions both in the steady state and in disease. In the gut, where the microbial load is greater than in any other body site, microbiota components constitute a continuous source of stimuli to which the immune system has evolved tolerance, leading to the modulation of immune responses. ^1^ Co-evolution with the myriad of microbes present in the gut has led to an equilibrium between the regulation of homeostatic responses to harmless antigens, and the ability to effectively eliminate pathogens. Although the role of adaptive immune cells in the gut has been extensively described, ^2^ the regulation of mononuclear phagocytic function (i.e. macrophages and dendritic cells) by microbiota members remains poorly understood. ^3^ Importantly, gut mononuclear phagocytes show aberrant anti-inflammatory responses in antibiotic-treated mice. These cells fail to regulate T cell populations in this organ, ^4^ while some bacterial species such as *Helicobacter hepaticus* ^5^ or *Clostridium butyricum* ^6^ directly contribute to IL-10 homeostasis. Remarkably, small populations of gut bacteria have been found to be associated with dendritic cells in the mesenteric lymph nodes, inducing the production of specific secretory IgA and promoting anti-inflammatory responses, including the release of the immunoregulatory cytokine IL-10. ^7^ Additionally, microbial metabolites, such as short-chain fatty acids (SCFAs), are known to regulate both dendritic cell and macrophage function in the gut, promoting anti-inflammatory/hyporesponsive states in innate immune cells and contributing to the development of intestinal homeostasis. ^3^

Since the discovery of phagocytic cells by Ilya Mechnikov, ^8^ our knowledge on monocyte and macrophage plasticity has enormously evolved. This includes phenomena described in the last decade such as the ability of different innate immune cell types, monocytes/macrophages among them, to generate long-term responses, namely innate immune memory. ^9^ This mechanism involves an epigenetic and metabolic reprogramming induced by the contact with an infectious agent or microbial component that leads to an enhanced (trained innate immunity) or decreased (tolerance) cytokine-mediated response to a secondary stimulation with a, usually different, microbial component. ^10,11^ Consequently, innate immune memory has been proven to be an important regulator not only of antimicrobial responses of innate immune cells, but of their roles in inflammatory and neurological diseases. ^12^ However, since all of these studies have been performed in a pathological context, no information is available to date regarding the role that innate immune memory plays in coping with the continuous exposure of innate immune cells to commensal microbes (i.e. human microbiota), and whether this phenomenon plays a role in immune modulation by commensal bacteria. ^13^

*Lactobacillus plantarum*, recently reclassified as *Lactiplantibacillus plantarum*, ^14^ is a Gram-positive species that has been extensively characterized for its adaptive ability to thrive within the human gut and the benefits of its immunomodulatory properties for the human host, with some *L. plantarum* strains being widely used as probiotics. ^15^ Therefore, we used this species as a model of a beneficial microbe to show that, while acute exposure to monocyte/macrophages allows bacterial intracellular survival and induce both pro- and anti-inflammatory cytokine release, a previous contact with *L. plantarum* reprograms the transcriptional and metabolic profiles of immune cells in the long term. This innate immune memory-like events eventually lead to enhanced bacterial intracellular survival and decreased pro-inflammatory features in pre-stimulated cells.

## Results

### Lactiplantibacillus plantarum *can survive within macrophages from different origins*

Recent reports have shown that dendritic cells populating the mesenteric lymph nodes harbor small amounts of gut bacteria inside, which contribute to the modulation of both innate and adaptive responses. ^7^ Given its genomic similarity with some intracellular pathogens, such as *Listeria monocytogenes*, ^16^ we hypothesized that *L. plantarum* might also be able to survive intracellularly and that this could be associated with its immunomodulatory effects. To unveil the potential of this species to survive intracellularly in macrophages we used antibiotic protection assays (Fig. S1A). As controls, we also used the closely related species, *Lactobacillus casei* (recently reclassified as *Lacticaseibacillus casei*) as well as the enteric species, *Escherichia coli*. First, using a multiplicity of infection (m.o.i.) of 10, we observed that all the *L. plantarum* strains used in this study were able to survive inside the macrophage-like cell line, RAW264.7 for 24 h, with strains of human milk origin showing slightly higher survival rates. In contrast, the number of *L. casei* and *E. coli* inside these phagocytes was lower (Fig. S1B).

**Figure 1.**
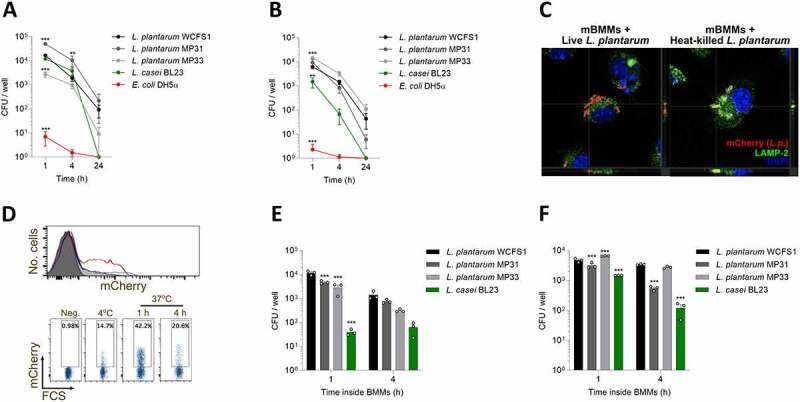
***L. plantarum* colonize and survive within macrophages from different origins**. Bacteria were co-cultured with either mouse bone marrow-derived macrophages (mBMMs) (a) or human monocyte-derived macrophages (hMDMs) (b), and their intracellular survival at different time points was determined using antibiotic protection assays (Fig. S1A). After incubating bacteria with immune cells, wells were washed, and antibiotic-containing medium was added. After 1, 4 or 24 h cells were lysed, and suspensions plated to assess bacterial intracellular survival. (c) Distinct colocalization of live (left micrograph) and heat-killed, mCherry-expressing *L. plantarum* (right micrograph, red) with the phagolysosome marker, LAMP-2 (green). The nuclei were stained with DAPI (blue). The orthogonal projection of the indicated points (crosshairs) is presented at the bottom and right side of the micrographs. (d) Anticoagulated whole blood was incubated with mCherry-labeled *L. plantarum* WCFS1 and its association with CD14^+^ cells was determined by flow cytometry. Representative flow cytometry data is shown. (e, f) Bacterial ability to persist intracellularly (e) and reach the extracellular medium (f) after being engulfed by immune cells. After short incubation times (1 and 4 hours) in antibiotic-containing medium, cells were extensively washed and incubated with antibiotic-free medium for 24 h. Samples from the supernatant and immune cell lysates were plated to check bacterial viability. Data are shown as mean ± s.e.m., n ≥ 3. **; *p* < .01, ***; *p* < .001, two-way ANOVA compared to *L. plantarum* WCFS1

In order to validate our results, we used two primary cellular models, murine bone marrow-derived macrophages (mBMM) and human monocyte-derived macrophages (hMDM). Overall, intracellular survival of *L. plantarum* strains was observed in both cell types, although to a lesser extent in comparison with the RAW264.7 cell line (Figure 1a, b). Similar to our observations using the cell line, intracellular survival of *L. casei* was lower than that of *L. plantarum* strains, with the presence of viable *E. coli* within the macrophages being dramatically decreased from the beginning of the experiment. Similar results were observed when an m.o.i. of 1 was used (Fig. S1C, D). To detect the cellular compartment in which *L. plantarum* persists inside macrophages, we incubated mBMMs with mCherry-labeled bacteria and assessed their colocalization with LAMP-2, a phagolysosome marker ^17^, using confocal microscopy. Notably, while heat-killed bacteria were observed surrounded by LAMP-2 positive vesicles, live bacteria did not show colocalization with the marker suggesting that they are able to evade their localization within these degradative organelles (Figure 1c).

We also performed whole blood *ex vivo* infection assays and analyzed the presence of bacteria. We incubated EDTA-treated human blood from healthy donors with *L. plantarum* WCFS1 modified to constitutively express mCherry and evaluated the internalization of the bacteria and its presence over time by flow cytometry. *L. plantarum* was found predominantly associated with CD14^+^ monocytes (Figure 1d). In fact, up to 42.2% of these cells were able to internalize and maintain *L. plantarum* for the duration of the assay.

Next, we assessed the ability of *L. plantarum* to escape from macrophages after its internalization. After adding bacteria and promoting phagocytosis for 45 min, extracellular bacteria were washed away, and macrophages were cultured either for 1 or 4 h in medium supplemented with antibiotics to kill remaining extracellular microbes (Fig. S1E). Then, mBMMs were washed with warm PBS and incubated in antibiotic-free medium for 24 h. Samples from supernatants and cell lysates were plated onto MRS agar plates to assess bacterial viability. Notably, high numbers of viable bacteria were found both in the intracellular (Figure 1e) and extracellular (figure 1f) compartments. Overall, *L. plantarum* strains survived better in comparison with *L. casei* both inside and outside of mBMMs. Therefore, these data show that after colonization of macrophages, *L. plantarum* can escape from the phagocytes to the extracellular medium.

Together, these data show that *L. plantarum* can be internalized by murine and human monocyte/macrophages and survive for prolonged periods of time, compared to other commensal bacteria.

### *Pre-exposure of macrophages to* L. plantarum *enhances bacterial survival and decreases pro-inflammatory outputs*

**Figure 2.**
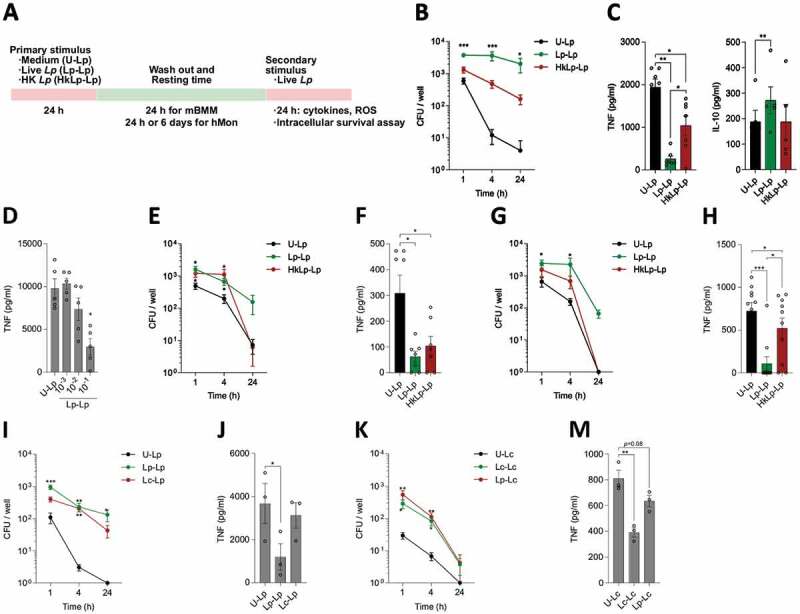
**Priming with *L. plantarum* enhances bacterial intracellular survival and reduces pro-inflammatory cytokine release**. (a) Diagram showing the experimental set up of priming experiments using murine bone marrow-derived macrophages (mBMMs) and human monocytes (hMon). Priming mBMMs with *L. plantarum* (m.o.i. = 1), especially in its live form, increased bacterial intracellular survival over time (b) and reduced the immune cell pro-inflammatory profile (c). (d) Decreased TNF release by primed cells depends on bacterial m.o.i. used. Intracellular survival and TNF production profiles showed similar patterns in primed hMon after either 24 h (e, f) or 6 d (g, h) resting time. Priming with either *L. plantarum* or *L. casei* improves bacterial survival (i, k) but does not reduce TNF release (j, m) if the other species is used for the second stimulation. Data are shown as mean ± s.e.m., n ≥ 3. *; *p* < .05, **; *p* < .01, ***; *p* < .001, two-way (B,E,G,I,K) or one-way (C,D,F,H,J,M) ANOVA

In the last decades, our knowledge on macrophage plasticity and ability to display long-term responses has greatly increased. Of note, research efforts have mainly focused on studying pathogenic/pathobiont microbes, partially ignoring the repertoire of long-lasting responses developed by these cells in response to different stimuli. ^18^ Thus, we assessed the ability of *L. plantarum* to induce innate immune memory-like responses in monocytes/macrophages *in vitro* (Figure 2a). Pre-stimulation of mBMMs with a m.o.i. of 1 of live *L. plantarum* (Lp-Lp) enhanced bacterial survival in a second encounter in comparison with unstimulated cells (U-Lp), while priming with heat-killed bacteria (HkLp-Lp) did not result in a significant increase in bacterial survival (Figure 2b). Of note, no bacterial survival was detected in the Lp-Lp group before the second stimulation, suggesting that live bacteria from the first stimulus had been eliminated. Remarkably, pre-stimulation with live bacteria also led to decreased TNF release after the second stimulation, while heat-inactivated bacteria promoted a more moderate reduction. In turn, cell culture supernatants of mBMMs primed with live bacteria contained increased levels of IL-10 compared to stimulated naïve cells (Figure 2c). This effect was not restricted to the WCSF1 strain as TNF decrease was also observed when using as priming agents two different strains isolated from human breast milk, MP31 (Fig. S2A) and MP33 (Fig. S2B). Notably, induction of memory-like features in mBMMs after *L. plantarum* exposure was dependent on bacterial dose, showing that a minimum of m.o.i. 0.1 used as the first stimulus was necessary to induce a significant decrease in TNF release upon a secondary stimulation (Figure 2d). Finally, to determine whether the induction of memory was due to secreted factors produced by *L*. plantarum, we pre-stimulated mBMMs with sterile MRS medium or filtered conditioned medium obtained from an overnight culture of *L. plantarum* WCFS1. No changes in intracellular survival of *L. plantarum* were observed (Fig. S2C), suggesting that soluble factors were not responsible for the increased survival of the bacterium upon stimulation with live microorganisms.

Our results were recapitulated in human CD14^+^ monocytes (hMon) *in vitro* using both 24 h or 6 d of resting time after the first stimulus, a protocol previously used to study induction of long-term responses in these cells. ^19^ While either live or heat-killed *L. plantarum* induced the same changes in bacterial intracellular survival and TNF production using a 24 h-resting time (Figure 2e and f), live *L. plantarum* induced higher effects when longer resting times were applied (Figure 2g and h). These changes in cytokine release profiles suggested that pre-stimulation with *L. plantarum*, particularly in its live form, induced long-term changes in both mBMMs and hMon featuring an anti-inflammatory profile, which may be involved in the increased bacterial intracellular survival observed.

We then tested whether priming with other probiotic bacterial species, *L. casei*, could lead to the same immunomodulatory events observed with *L. plantarum*. Of note, priming of mBMMs with live *L. casei* enhanced bacterial intracellular survival (Fig. S2D) and reduced TNF release (Fig. S2E) compared to unprimed cells, although to a lesser extent than when *L. plantarum* was used. Moreover, since trained cells have shown certain non-specificity in their responses to secondary challenges (e.g. BCG stimulation protects from fungal and bacterial infections ^12^), we tested the effect of priming with one probiotic species and use the other one as the secondary stimulation. Although trends of intracellular survival rates were comparable to those previously observed (Lp-Lp v. Lc-Lp, Figure 2i; Lc-Lc v. Lp-Lc, Figure 2k), priming with different species than those used for the second stimulation did not recapitulate the reduction in TNF levels (Figure 2j and m). These data show that for these bacteria, long-term effects on phagocytic cells are, at least partly, species-specific.

### L. plantarum *priming induces long-term changes in the transcriptional profile of human monocytes*

**Figure 3.**
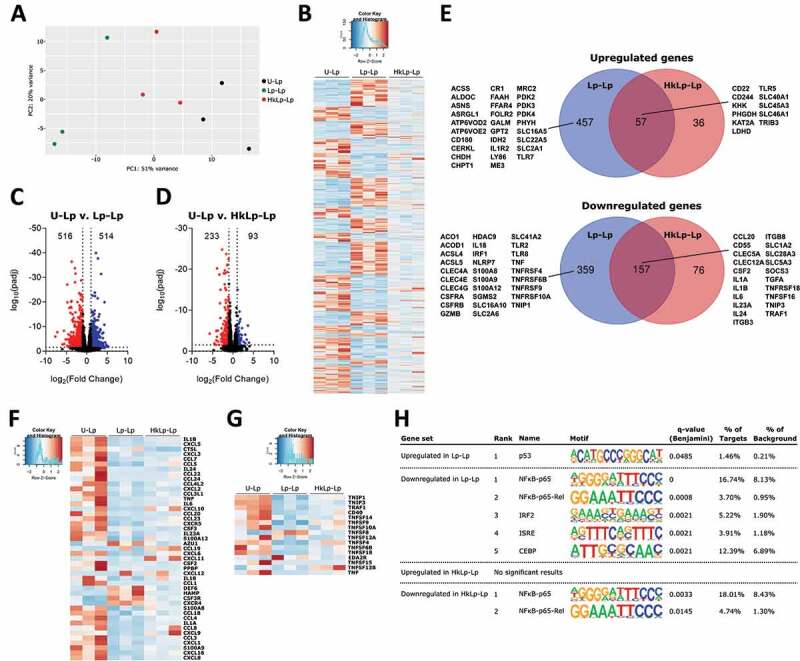
**Broad transcriptional remodeling is induced in human monocytes after *L. plantarum* priming**. (a) Principal Component Analysis of human monocytes (hMon) unprimed (U-Lp) or primed with either live (Lp-Lp) or heat-killed (HkLp-Lp) *L. plantarum*. Heat-map (b) and volcano plots (c, d) showing differentially regulated genes. Blue dots represent upregulated genes, whereas red dots indicate downregulated genes. (e) Venn diagrams depicting up- or down-regulated genes shared between Lp-Lp and HkLp-Lp compared to U-Lp. Heat-maps of selected differentially expressed genes involved in immune responses (f) and belonging to the TNF signaling pathway (g). (h) Comparative transcription factor enrichment analysis of differentially expressed genes using the HOMER package. The differential expression of genes was set at an absolute log_2_ Fold Induction value of 1 and Padj < 0.05

To delve into the mechanisms inducing these long-term responses by *L. plantarum*, we studied the transcriptional profiles of human monocytes by RNA-seq in the three experimental conditions previously analyzed after 6 d of resting time: U-Lp, Lp-Lp and HkLp-Lp. The three conditions showed distinct transcriptional profiles, as shown by principal component analysis (PCA) (Figure 3a) and clustering of the most regulated genes (Figure 3b). Overall, we found 1030 differentially expressed genes (using cut off values of 1 for the absolute log_2_ Fold Change and p adj <0.05) between unprimed and live bacteria-primed monocytes (U-Lp v. Lp-Lp, 514 up and 516 down; Figure 3c), and 326 when unprimed controls were compared with cells pre-stimulated with heat-killed bacteria (U-Lp v. HkLp-Lp, 93 up and 233 down; Figure 3d), showing that pre-exposure to live *L. plantarum* cells induced a greater impact on monocytes in the long term. Pathway analysis using PantherDB ^20,21^ (Table S1) showed a significant enrichment of several cytokine and chemokine pathways among the downregulated genes in monocytes primed either with live or heat-killed bacteria. Among others, genes as *IL1A, IL1B, IL6* or *CCL20* were found downregulated in both conditions compared to unstimulated cells (Figure 3e, f). In addition, pathway analysis showed downregulated functions related to organism killing and production of reactive oxygen and nitrogen species (Table S1). In this regard, several genes coding for antimicrobial peptides/proteins, such as calprotectin (*S100A8* and *S100A9*) and calgranulin (*S100A12*), were found downregulated only in the Lp-Lp group (Figure 3e, f), which may be linked to the increased bacterial intracellular survival observed in these cells. Notably, *TNF* and other six members of this cytokine signaling pathway were observed downregulated only in Lp-Lp monocytes, in addition to the four found in monocytes primed either with live or heat-killed bacteria (Figure 3g), which confirmed the profiles of TNF release previously detected by ELISA. Moreover, we found changes in the expression of *KAT2A* (upregulated in both Lp-Lp and HkLp-Lp) and *HDAC9* (downregulated in Lp-Lp), both of which are related to histone modifications and activation of transcription and might be involved in epigenetic modifications associated with innate immune memory.

Next, we carried out a comparative transcription factor enrichment analysis of the differentially expressed genes in Lp-Lp and HkLp-Lp using the HOMER package. ^22^ For upregulated genes, only the p53 motif was found as regulator of 1.46% of genes in the Lp-Lp condition, while no significant results were found for the HkLp-Lp gene set (Figure 3h). On the other hand, motifs associated with NFκB-p65 contributed to the highest percentage of downregulated genes in both live (Lp-Lp condition) and heat-killed (HkLp-Lp condition) bacteria-primed cells, with minor contributions from interferon-regulated transcription factors (IRF2, ISRE) and CBEP under live bacteria priming conditions (Figure 3h).

### *Transcriptional reprogramming in* L. plantarum*-stimulated monocytes induce changes in cell metabolism*

**Figure 4.**
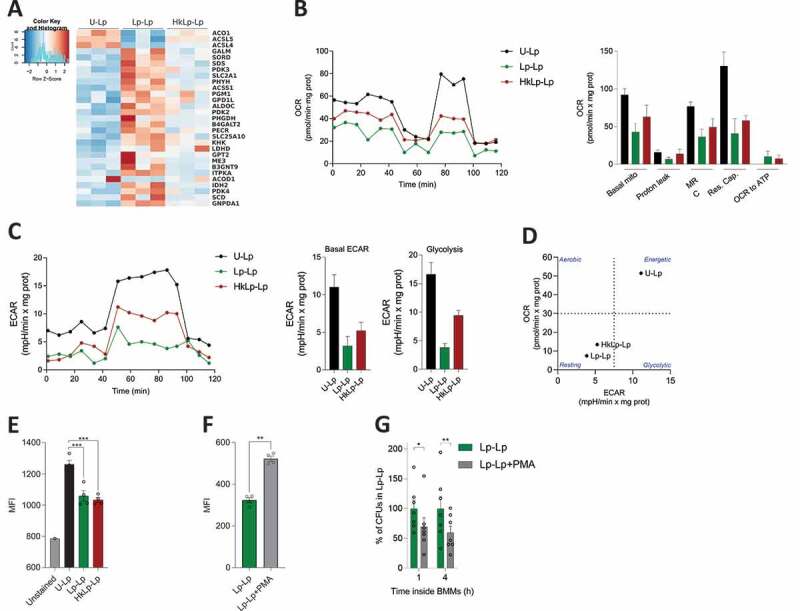
***L. plantarum* priming promotes a metabolic rewiring resulting in decreased oxidative burst**. (a) Heat-map depicting selected differentially expressed genes with functions related to cellular metabolism. Seahorse extracellular flux analyzer was used to determine OCR (b) and ECAR (c) profiles of human monocytes (hMon). Data are shown for a representative experiment out of two independently performed. (d) Phenogram showing OCR/ECAR ratio of unprimed hMon, and cells primed with either live (Lp-Lp) or heat-killed (HkLp-Lp) *L. plantarum*. (e) ROS production after the second bacterial encounter. Phorbol-12-myristate-13-acetate (PMA) was used to increase ROS production in mBMMs (f), which induced a decrease in *L. plantarum* intracellular survival in memory macrophages (g). Data are shown as mean ± s.e.m., n ≥ 3. *; *p* < .05, **; *p* < .01, ***; *p* < .001, One-way ANOVA (E) and Student’s t test (f,g)

Analysis of monocyte transcriptomic profiles also allowed us to identify the impact of priming in several metabolic pathways of monocytes in comparison with cells acutely exposed to *L. plantarum*, especially in those monocytes pre-stimulated with live bacteria (Figure 4a). Although we did not find great changes in the expression levels within members of central metabolic pathways, we observed that some adjacent metabolic pathways were enriched in our functional study. Indeed, our data showed the upregulation of folic acid metabolism, amino acid and carboxylic acid biosynthesis, and monocarboxylic acid catabolism, and the downregulation of hyaluronan biosynthesis, negative regulation of lipid storage, and glycerol transport (Table S1). Specifically, we observed the upregulation of three pyruvate dehydrogenase kinases genes (*PDK2, PDK3, PDK4*) in cells primed with live bacteria, as well as a decreased expression of *ACO1* and *ACOD1*, coding for aconitase and aconitate decarboxylase, suggesting a reduction in the integrity of the tricarboxylic acid (TCA) cycle and the itaconate pathway. We also found the differential regulation of several genes coding for metabolite transporters (Figure 3e), including those for glucose (*SLC2A1*), other hexoses and monocarboxylic compounds (*SLC2A6, SLC45A3, SLC16A5*), and amino acids (*SLC1A2, SLC16A10*), which possibly contribute to changes in cellular metabolism.

To assess whether these alterations in the transcriptional landscape had physiological consequences in primed cells, we analyzed the metabolic profiles of human monocytes by measuring their oxygen consumption rate (OCR) and extracellular acidification rate (ECAR) using the Seahorse extracellular flux analyzer. First, following the same experimental rationale previously used with the 6-d resting time (Figure 2a), we observed decreased levels in both OCR (Figure 4b) and ECAR (Figure 4c) in human monocytes pre-stimulated with either live or heat-killed *L. plantarum* compared to unprimed cells, with the metabolic alterations in the group Lp-Lp being slightly higher. Consequently, the phenogram comparing basal OCR and ECAR clearly showed the metabolic shift induced by monocyte priming with *L. plantarum*. While acutely stimulated cells showed high metabolic levels, memory monocytes exhibited a metabolic profile similar to that observed in resting cells (Figure 4d). Of note, these results were recapitulated in mBMMs, which showed decreased OCR (Fig. S3A), ECAR (Fig. S3B) and a clear separation between the three experimental groups in the phenogram, especially for Lp-Lp (Fig. S3C). In accordance with a decrease in the glycolytic activity, we did not detect any significant change in lactate production between experimental groups in either human monocytes (Fig. S3D) or mBMMs (Fig. S3E).

Reduction in mitochondrial respiration after induction of innate memory in monocytes/macrophages led us to hypothesize that priming cells with *L. plantarum* might be modulating reactive oxygen species (ROS) production, since the mitochondrial electron transport chain is one of the main sources of these antimicrobial molecules in macrophages. ^23^ In fact, both reactive oxygen (fold enrichment 4.04) and nitrogen species (fold enrichment 5.8) production pathways were found to be enriched in the PantherDB analysis of the downregulated set of genes in the Lp-Lp condition (Table S1). Thus, we used the mitochondrial superoxide staining MitoSOX to measure the oxidative stress in primed compared to acutely stimulated cells using mBMMs as a model. Remarkably, ROS production was significantly reduced in memory macrophages, either pre-stimulated with live or heat-killed bacteria (Figure 4e). To further analyze the role of ROS in the observed increase in intracellular survival induced by cell priming, we triggered ROS production in mBMMs previously exposed to live *L. plantarum* just before the second bacterial encounter by using phorbol-12-myristate-13-acetate (PMA). Increased production of ROS in PMA-treated cells (figure 4f) correlated with a reduction in bacterial intracellular survival of ~30% at 1 h and ~40% at 4 h (Figure 4g), evidencing the remarkable role of the decreased oxidative burst occurring in primed macrophages for *L. plantarum* persistence.

Overall, these results show that priming with *L. plantarum*, especially with live bacteria, leads to a metabolic reprogramming that results in decreased ROS production and the enhanced intracellular survival of bacteria in memory cells.

## Discussion

The discovery of the ability of innate immune cells to develop long-term responses, i.e. trained innate immunity or tolerance, challenged the classic immunology dogma of innate versus adaptive immunity. ^24^ Importantly, molecular mechanisms underlying these long-term responses have been, at least partly, elucidated in the last two decades, ^9,25–27^ increasing the possibility of improving, for example, vaccination strategies. ^28,29^ However, these studies have been only focused on analyzing the response of innate immune cells to pathogenic microbes, such as *Candida albicans* or *Mycobacterium tuberculosis*, almost exclusively using microbe-derived PAMPs or dead microbial cells. ^11^ Unfortunately, no attention has been paid to the role of this phenomenon in the interaction of the host with the myriads of beneficial microbial species inhabiting the human body, even though a huge plasticity of innate immune memory in response to diverse stimuli has been previously reported. ^18^

Modulation of innate immune responses by the gut microbiota occurs by different mechanisms. ^3^ These include the presence and physiological activities of resident intracellular bacteria in mononuclear phagocytes that are essential for inflammation control and homeostasis in the intestine. ^7^ Therefore, we first explored the ability of *L. plantarum*, a bacterial species containing well-characterized probiotic strains, to survive inside macrophages. Indeed, we observed that this species can survive for hours inside both murine and human macrophages, and that human CD14^+^ monocytes are able to carry these bacteria. Notably, *L. plantarum* intracellular survival was more restricted than that observed for other bacterial species with a more defined intracellular lifestyle, such as the closely related *L. monocytogenes*, ^30,31^ suggesting that this species has not developed a complete genetic machinery to thrive in the long term in this environment. However, we hypothesized that *L. plantarum* intracellular survival might play a major role in their immunomodulatory properties, and that previous encounters with these bacteria may induce long-term responses in innate immune cells.

Strikingly, pre-stimulation with *L. plantarum*, especially in its live form, significantly enhanced bacterial survival and decreased pro-inflammatory responses in mBMMs after a resting period of 24 h. To test whether these changes induced by cell priming had effects in the long term, we performed *in vitro* experiments using human monocytes as previously reported. ^19^ Notably, we observed similar results in human monocytes regarding intracellular survival and TNF release, with a more pronounced effect found when cells were left resting for 6 d. Although to a lesser extent, we showed that *L. casei* was able to induce similar effects as *L. plantarum* did and that induction of this tolerogenic type of innate immune memory leads to a certain degree of cross-reactivity. The role of gut microbiota in the modulation of intestinal macrophage function is critical for gut homeostasis, ^3,32,33^ yet poorly understood. Our data suggest that prolonged contact with some members of the gut microbiota, at least *L. plantarum* and *L. casei* species, induces a long-term anti-inflammatory state in monocytes/macrophages that expands the immunomodulatory properties already described for these species. ^34,35^ Human monocytes have been routinely studied for the development of innate memory. Unlike other tissue-resident macrophage populations, which have embryonic origin, intestinal macrophages originate primarily from circulating monocytes ^36^, and their recruitment to the gut is partially mediated by microbiota-derived signals ^37^. This makes blood monocytes a good model to analyze the cellular and molecular events during the interaction between gut microbiota and phagocytes.

Our results show that the response to live microorganisms differs from those observed when killed forms of the same microorganism are used, as exemplified for other bacteria such as *Salmonella* ^38^ or *Borrelia burgdorferi* ^39^. The difference is likely linked to either alterations in cell wall composition/structure or the lack of microbial physiological activities leading to the production of metabolites, proteins, etc. that may activate innate immune cells. Lactobacilli produce a wide array of proteins and metabolites, such as short-chain fatty acids, ^40,41^ that are secreted to the extracellular medium and might exert immunomodulatory properties.

Overall, our results contrast with previous reports focused on innate immune memory development, either training or tolerance, using pathogen-derived PAMPs, such as LPS, β-glucan or BCG. ^12,42,43^ In fact, the major PAMPs that can be found in *L. plantarum*, peptidoglycan, lipoteichoic acid and flagellin, have been previously associated with enhanced memory responses (i.e. trained immunity) when only cytokine production (TNF and IL-6) responses where considered. ^18^ However, this effect was shown to be dose-dependent and since we used whole bacterial cells, integration of different cell signals originating from the entire microbe led to a different phenotype. More importantly, our experiments comparing memory induction using live or heat-killed bacteria showed a strong influence of bacterial viability in most of the aspects analyzed. Therefore, besides the relevance of classical PAMPs, our data strongly suggest that other factors secreted/produced by live bacteria are critical for innate memory development. Priming of mBMMs with conditioned medium did not, however, induced a memory phenotype, suggesting that physiological activities other that secreted factors contribute to the development of innate memory in a dose-dependent manner.

Transcriptomic analyses of primed human monocytes further confirmed the prominent decrease in the expression of pro-inflammatory mediators and effectors, including an array of cytokines, chemokines and antimicrobial peptides. Moreover, we observed a differential regulation of genes and pathways involved in metabolism, especially in the transport and use of carbohydrates, amino acids and fatty acids, which could be linked to the decreased metabolic rates and ROS production observed in primed cells. Cellular metabolism is intimately linked to the regulation of immune responses, with metabolic rewiring being described in both acutely stimulated ^44^ and innate memory cells ^45^. Notably, priming with live bacteria increased the expression of pyruvate dehydrogenase kinase genes *PDK2, PDK3, PDK4*. These proteins are involved in cellular metabolism regulation through the inactivation of components of pyruvate dehydrogenase, the enzyme complex converting pyruvate to acetyl-CoA, leading to decreased glucose and lipid metabolism, and aerobic respiration. ^46^ In addition, *ACO1* and *ACOD1*, coding for aconitase and aconitate decarboxylase, were found downregulated. Itaconate, a metabolite with anti-microbial and immunomodulatory properties, ^47^ has been associated with the modulation of β-glucan-induced trained immunity, and *ACOD1* expression showed decreased levels in memory monocytes compared to acutely stimulated cells. ^48^ Thus, the observed differential gene expression suggests a reduction in the integrity of the tricarboxylic acid (TCA) cycle and the itaconate pathway that might be relevant for the *L. plantarum*-induced long-term memory phenotype in monocytes.

Further physiological studies confirmed the metabolic alterations in *L. plantarum*-primed cells identified by transcriptomic analyses, with primed phagocytes showing a drop in glycolytic and respiratory rates and shifting to a “resting-like” phenotype. These data strongly contrast with previous reports using pathogen cells or PAMPs. Monocyte/macrophages acutely stimulated with LPS, ^44^ or primed for long-term experiments with LPS, ^49^ β-glucan ^50^ or BCG ^51^ exhibit increased glycolytic rates, with oxygen consumption being more variable between conditions. Also, we found that decreased mitochondrial respiration was coupled with a reduced ROS production in cells primed with *L. plantarum* after receiving a second stimulation. Lower ROS production has been also reported in β-glucan-trained monocytes, which also exhibit reduced OCR, ^50^ but contrasts with the phenotypes observed in cells primed with BCG or oxidized LDL. ^19^ Of note, rescuing the oxidative burst using PMA reduced bacterial survival, suggesting a link between the immunometabolic changes observed in *L. plantarum*-induced memory cells and their tolerogenic anti-inflammatory phenotype.

Taken together, our results show the capacity of commensal and probiotic species, such as *L. plantarum*, to survive intracellularly in a, in principle, hostile environment. These data also establish the ability of symbiotic bacteria to induce long-term memory responses in innate immune cells through mechanisms that involve the metabolic rewiring of phagocytic cells and the decreased induction of deleterious antibacterial compounds, such as ROS. Thus, our observations suggest that *L. plantarum*-mediated induction of innate immune memory may contribute to inflammation control and to the expansion of these bacteria into alternative ecological niches such as the immune cell intracellular compartment. Further studies should delineate the in vivo effects of innate immune memory induction by symbiotic microorganisms and the contribution of these interactions on gut homeostasis.

## Experimental procedures

### Ethics statement

All procedures and experiments involving animals, including their housing and care, were carried according to the guidelines of the European Union Council (Directive 2010/63/EU) and Spanish Government regulations (RD 53/2013), and with the approval of the ethics committee of CIC bioGUNE and the Competent Authority (Diputación de Bizkaia). The Animal Facility at CIC bioGUNE is accredited by AAALAC Intl.

Buffy coats from healthy blood donors were obtained from the Basque Biobank, after approval by the Basque Country’s Ethics committee following the Helsinki convention. Donors had a median age of 53 (26–72) and were 68% male, 32% female (n = 37).

### Bacterial strains and growth conditions

In this study, we used *L. plantarum* strain WCFS1, isolated from human saliva ^16^ and two strains isolated from human breast milk, MP31 and MP33. ^52^ In addition, *L. casei* BL23 and *Escherichia coli* DH5α were assessed for their intracellular survival. All strains were kept frozen at −80°C and thawed as needed. *L. plantarum and L. casei* strains were grown statically in De Man-Rogosa-Sharpe (MRS) medium at 37°C, while *E. coli* was cultivated in Luria-Bertani (LB) at 37°C and 200 rpm until they reached the logarithmic phase of growth (O.D ≈ 0.6). All strains used in this study were shown to be sensible to penicillin-streptomycin.

In order to calculate the multiplicity of infection (m.o.i.) before intracellular survival assays, an estimation of bacterial numbers in culture was performed. A correlation equation was calculated specifically for each strain, using for that purpose the association between culture optical density at 600 nm and colony forming units determined by dilution plating. Heat-killed bacteria were obtained by incubating bacterial suspensions in a water bath at 70°C for 15 min.

### Mammalian cell culture

The macrophage-like murine cell line RAW264.7 was maintained in DMEM (Lonza, Spain) supplemented with 10% FBS and 1% penicillin-streptomycin. Mouse bone marrow-derived macrophages (mBMMs) were generated from 8–12-week-old C57Bl/6 (B6) mice as previously described. ^53^ Briefly, bone marrow cells were flushed out from clean femurs and tibias, filtered through a 70 µm-nylon mesh (Thermo Fisher Scientific, Rockford, IL, USA), and centrifuged at 400 xg for 5 min. Red blood cells were removed using ACK lysis buffer, and the remaining cells were incubated in 100 mm × 15 mm Petri dishes for 7 d in DMEM with 10% FBS and 1% penicillin-streptomycin plus 30 ng/ml of M-CSF (Miltenyi Biotec, Bergisch Gladbach, GE). Fresh medium was added after 3 d of culture.

To obtain human CD14^+^ monocytes, cell suspensions from buffy coats were placed onto a Ficoll layer and centrifuged at 400 xg for 30 min without brake. The layer corresponding to peripheral blood monocytic cells was obtained and monocytes were positively selected using a human CD14 positive cells purification kit (Miltenyi Biotec) following the manufacturer’s instructions. To generate human monocyte-derived macrophages (hMDMs), purified monocytes were incubated for 7 d in RPMI 1640 medium (Lonza) supplemented with 10% FBS, 2.4 mM L-glutamine, 1% penicillin–streptomycin and 30 ng/ml human M-CSF (Miltenyi Biotec). The medium was refreshed 3 d after extraction.

### Antibiotic protection assays

The ability of the bacterial strains to survive inside mammalian macrophages was assessed by antibiotic protection assays. ^54^ RAW 264.7, mBMMs and hMDMs were seeded onto 24-well plates at a density of 2 × 10^5^ cells per well. Due to sample size, experiments involving human cells were performed in 96-well plates using 2 × 10^4^ cells per well. After 24 h, cells were extensively washed with warm PBS and bacteria were added in serum- and antibiotic-free DMEM. The cultures were synchronized by an incubation of 30 min at 4°C and then, plates were incubated for 45 min at 37°C to promote phagocytosis. Extracellular bacteria were then washed with pre-warmed PBS, and those remaining were eliminated by a 1-hour incubation with serum- and antibiotic-supplemented DMEM. Phagocytes were lysed at different timepoints in DMEM/0.1% Triton X-100, and lysates seeded onto MRS or LB agar plates for colony forming unit (CFU) quantification. Samples from the supernatant were also seeded to test the absence of extracellular bacteria after the incubation with antibiotics.

To assess the ability of *L. plantarum* WCFS1 to extrude from phagocytes, bacteria were added as previously detailed and those remaining extracellularly eliminated by extensive washing and addition of antibiotics. After one or 4 hours, the medium was replaced with antibiotic-free DMEM and cultures were incubated at 37°C for 24 h. Then, samples from the supernatants and the cell lysates were plated for CFU enumeration.

### *Detection of subcellular localization of* L. plantarum

To detect whether *L. plantarum* was contained inside phagolysosomes in macrophages, we incubated mBMMs and mCherry-labeled *L. plantarum* cells, either live or heat-killed, in 24-well plates containing sterile round coverslips. After the incubation time (30 min at 4°C, 45 min at 37°C, and 1 h in the presence of antibiotics), cells were washed twice with PBS and fixed with 4% PFA for 30 min at RT. The wells were washed with PBS and blocked with PBS/0.3% triton X-100/1%BSA/0.25 M NaCl for 1 h at RT. The lysosome-associated membrane protein 2 (LAMP-2), a marker for phagosomes and phagolysosomes,^55^ was detected with a monoclonal rat anti-mouse LAMP-2 primary antibody (Abcam; ab13524) at 4 µg/ml in PBS/0.3% triton X-100 overnight at 4°C. Wells were washed three times with PBS/0.3% triton X-100, 10 min each, and incubated with anti-rat Alexa Fluor 488 (Invitrogen; A-11006) at 4 µg/ml in PBS/0.3% triton X-100 for 1 h at RT. The wells were then washed three times with PBS/0.3% triton X-100, 10 min each, including DAPI nuclei staining in the last washing step. The coverslips were washed three times with PBS and prepared for confocal microscopy using Prolong Gold Antifade reagent. Micrographs were obtained employing a Leica TCS SP8 confocal system (Leica Microsystems, Madrid, Spain).

### Whole blood infection model

Human blood samples from healthy donors were incubated with EDTA to prevent coagulation, and *L. plantarum* WCFS1 expressing mCherry was added, as previously described. ^56^ After incubation at either 4°C or 37°C, samples were stained with anti-human CD14-APC for 30 min in ice. After washing samples with PBS/1% fetal calf serum, erythrocytes were eliminated using ACK buffer, and the presence of fluorescent bacteria inside CD14^+^ monocytes was detected by flow cytometry.

### Cytokine and ROS measurements

Levels of TNF and IL-10 in the murine or human cell supernatants were determined by ELISA using the Mouse TNF ELISA Set II, the Mouse IL-10 ELISA set (BD Biosciences) and the human TNF ELISA set (Thermo Fisher Scientific), following the manufacturer’s instructions.

ROS production by memory mBMMs was assessed using MitoSOX Red (Life Technologies). Briefly, after stimulation experiments, cells were washed and stained with MitoSOX Red for 30 min at 37°C. After extensive washing with warm PBS, cells were trypsinized and fluorescence signal was acquired by flow cytometry in a BD FACS Canto II cytometer (BD Biosciences, Madrid, Spain). Phorbol-12-myristate-13-acetate (PMA) was used at 0.8 μM to increase ROS in memory macrophages 30 min prior to the second incubation with *L. plantarum*.

### RNA extraction and RNA-seq

Total RNA samples were obtained using the NucleoSpin® RNA kit (Macherey-Nagel). The quantity and quality of the RNAs were assessed using the Qubit RNA Assay Kit (Thermo Fisher Scientific) and RNA Nano Chips in a 2100 Bioanalyzer (Agilent Technologies), respectively.

Libraries were prepared using the NuGEN Universal Plus mRNA-Seq kit (Tecan Genomics Inc., NuGEN) following the manufacturer’s instructions. Single-read 50 nt sequencing of pooled libraries was carried out in a HiSeq2500 platform (Illumina). Quality control of sequenced samples was performed utilizing the FASTQC software (http://www.bioinformatics.babraham.ac.uk/projects/fastqc/). Reads were mapped against the human (hg38) reference genome using the STAR program ^57^ to account for spliced junctions. The resulting BAM alignment files for the samples were used to generate a table of raw counts using Rsubread. ^58^ A raw counts table was the input for the Differential Expression analysis performed with DESeq2, ^59^ to compare the different conditions. Heatmaps were plotted via heatmap.2 function from the gplots R package (https://CRAN.R-project.org/package=gplots), using a regularized log transformation (RLB) on DESeq2 normalized reads in order to enhance graphical representation of the data. Venn diagrams were produced using the tool developed by Van de Peer (http://bioinformatics.psb.ugent.be/webtools/Venn/). GO enrichment was tested using PantherDB. ^20,21^

Motif enrichment analysis was performed using HOMER (v4.10.4). The “findMotifs.pl” wrapper script ^22^ was used for de novo motif discovery (using the following parameters: human as reference genome and 8, 10, 12 pbs as motif lengths) to check the enrichment of known and *de novo* motifs. The input corresponded to the list of gene IDs differentially expressed in the RNA-seq analysis for each of the comparatives analyzed.

### Metabolic profiling

Oxygen consumption (OCR) and extracellular acidification (ECAR) rates were measured in differentially stimulated murine macrophages or human monocytes in an XF24 extracellular flux analyzer (Agilent). Unstimulated and *L. plantarum*-stimulated cells (2.5–5 × 10^5^ per well) were seeded in a Cell-Tak coated plate (BD Biosciences). The measurements were normalized to cellular protein amount. For ECAR determination, the cells were previously plated in XF Seahorse medium with 4 mM glutamine and 10 mM pyruvate, while for the mitochondrial stress test the cells were plated in medium containing 4 mM glutamine, 10 mM pyruvate and 25 mM glucose. After 1 h at 37°C without CO_2_, three baseline oxidative consumption rate (OCR) and extracellular acidification rate (ECAR) measurements were performed. For glycolysis determination, ECAR was measured at baseline and after sequentially adding glucose (25 mM), Oligomycin (1 µM) and 2-DG (50 mM). In parallel experiments, OCR was determined at baseline and after sequentially adding oligomycin, FCCP, antimycin/rotenone at 1 µM.

Lactate production was measured from stimulation supernatants using the Lactate-Glo™ Assay kit (Promega) following the manufacturer’s instructions.

### Statistics

Statistical analyses were performed using GraphPad Prism (GraphPad Software Inc., CA, USA). At least three biological replicates were performed to measure each parameter in each experimental condition.

## Supplementary Material

Supplemental Material

## Data Availability

The authors confirm that the data supporting the findings of this study are available within the article and its supplementary materials. The accession number for the RNAseq data is NCBI GEO: GSE159496.
