# Supplementary material for: The commensal bacterium Lactiplantibacillus plantarum imprints innate memory-like responses in mononuclear phagocytes
Source: Gut Microbes. 2021 Jul 5;13(1):1939598. doi: 10.1080/19490976.2021.1939598 (PMC8259724; doi:10.1080/19490976.2021.1939598)
Supplement: Supplemental Material [file KGMI_A_1939598_SM6562.zip › supplementary/Table S1.docx]

**Table S1.** Enriched PantherDB pathways of genes differentially expressed in primed monocytes (Lp-Lp or HkLp-Lp) compared to unprimed controls (U-Lp).

| **GO terms associated with upregulated genes in Lp-Lp compared to U-Lp** | | | | | |
| --- | --- | --- | --- | --- | --- |
| **GO biological process complete** | **Client Text Box Input (fold Enrichment)** | **Client Text Box Input (raw P-value)** | **Client Text Box Input (FDR)** | **Client Text Box Input (482)** |  |
| Folic acid metabolic process (GO:0046655) | 13.76 | 1.53E-05 | 8.09E-03 | 6 |  |
| G protein-coupled purinergic receptor signaling pathway (GO:0035588) | 10.89 | 2.04E-04 | 4.06E-02 | 5 |  |
| Cellular modified amino acid biosynthetic process (GO:0042398) | 7.41 | 2.93E-05 | 1.37E-02 | 8 |  |
| Positive regulation of myeloid leukocyte differentiation (GO:0002763) | 6.01 | 1.11E-04 | 2.81E-02 | 8 |  |
| Regulation of protein processing (GO:0070613) | 5.68 | 6.19E-05 | 1.89E-02 | 9 |  |
| Monocarboxylic acid catabolic process (GO:0072329) | 4.2 | 1.21E-04 | 2.87E-02 | 11 |  |
| Negative regulation of peptide secretion (GO:0002792) | 3.9 | 1.16E-04 | 2.88E-02 | 12 |  |
| Regulation of cellular carbohydrate metabolic process (GO:0010675) | 3.53 | 2.73E-04 | 4.88E-02 | 12 |  |
| Carboxylic acid biosynthetic process (GO:0046394) | 3.05 | 1.34E-05 | 8.50E-03 | 21 |  |
| Response to peptide (GO:1901652) | 2.37 | 9.03E-05 | 2.48E-02 | 26 |  |
| Carbohydrate metabolic process (GO:0005975) | 2.36 | 9.27E-05 | 2.50E-02 | 26 |  |
| Regulation of neurogenesis (GO:0050767) | 2.05 | 4.97E-05 | 1.79E-02 | 39 |  |
| Regulation of protein kinase activity (GO:0045859) | 1.98 | 1.64E-04 | 3.38E-02 | 36 |  |
| Negative regulation of molecular function (GO:0044092) | 1.94 | 8.81E-06 | 6.67E-03 | 52 |  |
| Leukocyte activation (GO:0045321) | 1.9 | 1.84E-04 | 3.75E-02 | 40 |  |
| Regulation of response to external stimulus (GO:0032101) | 1.88 | 5.23E-05 | 1.77E-02 | 47 |  |
| Cellular response to oxygen-containing compound (GO:1901701) | 1.83 | 1.58E-04 | 3.39E-02 | 45 |  |
| Positive regulation of phosphate metabolic process (GO:0045937) | 1.8 | 1.59E-04 | 3.33E-02 | 47 |  |
| Positive regulation of cellular protein metabolic process (GO:0032270) | 1.73 | 3.22E-05 | 1.46E-02 | 64 |  |
| Response to external stimulus (GO:0009605) | 1.66 | 1.46E-06 | 1.93E-03 | 93 |  |
| Positive regulation of signal transduction (GO:0009967) | 1.65 | 1.36E-04 | 3.01E-02 | 64 |  |
| Regulation of biological quality (GO:0065008) | 1.49 | 9.47E-07 | 1.37E-03 | 139 |  |
| Response to stress (GO:0006950) | 1.42 | 6.86E-05 | 1.95E-02 | 117 |  |
| Unclassified (UNCLASSIFIED) | 0.5 | 7.37E-07 | 1.30E-03 | 36 |  |
| RNA processing (GO:0006396) | 0.2 | 3.25E-05 | 1.40E-02 | 4 |  |
| Mrna metabolic process (GO:0016071) | 0.06 | 3.13E-06 | 2.93E-03 | 1 |  |
| Detection of chemical stimulus involved in sensory perception of smell (GO:0050911) | 0.01 | 9.88E-05 | 2.53E-02 | 0 |  |
|  |  |  |  |  |  |
|  |  |  |  |  |  |
| **GO terms associated with downregulated genes in Lp-Lp compared to U-Lp** | | | | | |
| **GO biological process complete** | **Client Text Box Input (fold Enrichment)** | **Client Text Box Input (raw P-value)** | **Client Text Box Input (FDR)** | **Client Text Box Input (482)** |  |
| regulation of platelet-derived growth factor production (GO:0090361) | 44.48 | 2.01E-04 | 7.70E-03 | 3 |  |
| positive regulation of natural killer cell chemotaxis (GO:2000503) | 29.66 | 4.36E-05 | 2.06E-03 | 4 |  |
| positive regulation of platelet-derived growth factor receptor signaling pathway (GO:0010641) | 26.69 | 5.45E-04 | 1.82E-02 | 3 |  |
| regulation of calcidiol 1-monooxygenase activity (GO:0060558) | 25.42 | 6.73E-05 | 3.03E-03 | 4 |  |
| eosinophil chemotaxis (GO:0048245) | 20.02 | 7.54E-09 | 6.77E-07 | 9 |  |
| hyaluronan biosynthetic process (GO:0030213) | 19.06 | 1.13E-03 | 3.28E-02 | 3 |  |
| glycerol transport (GO:0015793) | 19.06 | 1.13E-03 | 3.29E-02 | 3 |  |
| positive regulation of fever generation (GO:0031622) | 19.06 | 1.13E-03 | 3.30E-02 | 3 |  |
| positive regulation of transforming growth factor beta1 production (GO:0032914) | 19.06 | 1.13E-03 | 3.30E-02 | 3 |  |
| chemokine production (GO:0032602) | 17.79 | 1.94E-04 | 7.48E-03 | 4 |  |
| positive regulation of granulocyte macrophage colony-stimulating factor production (GO:0032725) | 17.11 | 3.40E-05 | 1.62E-03 | 5 |  |
| CD4-positive, alpha-beta T cell cytokine production (GO:0035743) | 16.68 | 1.53E-03 | 4.12E-02 | 3 |  |
| L-glutamate import across plasma membrane (GO:0098712) | 16.68 | 1.53E-03 | 4.14E-02 | 3 |  |
| positive regulation of corticosteroid hormone secretion (GO:2000848) | 16.68 | 1.53E-03 | 4.15E-02 | 3 |  |
| regulation of neutrophil apoptotic process (GO:0033029) | 16.68 | 1.53E-03 | 4.15E-02 | 3 |  |
| urea transmembrane transport (GO:0071918) | 16.68 | 1.53E-03 | 4.16E-02 | 3 |  |
| leukocyte aggregation (GO:0070486) | 16.18 | 2.60E-04 | 9.65E-03 | 4 |  |
| positive regulation of podosome assembly (GO:0071803) | 16.18 | 2.60E-04 | 9.67E-03 | 4 |  |
| monocyte chemotaxis (GO:0002548) | 15.89 | 1.03E-12 | 1.82E-10 | 15 |  |
| positive regulation of neuroinflammatory response (GO:0150078) | 15.89 | 4.53E-05 | 2.13E-03 | 5 |  |
| neutrophil chemotaxis (GO:0030593) | 15.01 | 4.68E-22 | 5.32E-19 | 28 |  |
| chronic inflammatory response (GO:0002544) | 13.69 | 4.37E-04 | 1.51E-02 | 4 |  |
| lymphocyte chemotaxis (GO:0048247) | 13.62 | 6.47E-12 | 9.89E-10 | 15 |  |
| positive regulation of interleukin-17 production (GO:0032740) | 13.08 | 9.72E-05 | 4.17E-03 | 5 |  |
| chemokine-mediated signaling pathway (GO:0070098) | 12.79 | 4.59E-17 | 1.62E-14 | 23 |  |
| positive regulation of nitric-oxide synthase biosynthetic process (GO:0051770) | 11.86 | 6.88E-04 | 2.21E-02 | 4 |  |
| positive regulation of T-helper 1 type immune response (GO:0002827) | 11.71 | 1.51E-04 | 6.12E-03 | 5 |  |
| negative regulation of morphogenesis of an epithelium (GO:1905331) | 11.12 | 8.45E-04 | 2.58E-02 | 4 |  |
| positive regulation of monocyte chemotaxis (GO:0090026) | 10.59 | 2.26E-04 | 8.55E-03 | 5 |  |
| ovulation (GO:0030728) | 10.47 | 1.03E-03 | 3.03E-02 | 4 |  |
| positive regulation of humoral immune response (GO:0002922) | 10.47 | 1.03E-03 | 3.03E-02 | 4 |  |
| regulation of interferon-gamma biosynthetic process (GO:0045072) | 10.47 | 1.03E-03 | 3.04E-02 | 4 |  |
| regulation of immunoglobulin secretion (GO:0051023) | 9.89 | 1.23E-03 | 3.51E-02 | 4 |  |
| positive regulation of tyrosine phosphorylation of STAT protein (GO:0042531) | 9.81 | 3.36E-10 | 3.88E-08 | 15 |  |
| negative regulation of lipid storage (GO:0010888) | 9.36 | 1.47E-03 | 4.02E-02 | 4 |  |
| cellular response to fluid shear stress (GO:0071498) | 9.36 | 1.47E-03 | 4.03E-02 | 4 |  |
| extracellular matrix assembly (GO:0085029) | 9.2 | 1.03E-04 | 4.37E-03 | 6 |  |
| response to vitamin D (GO:0033280) | 9.2 | 1.03E-04 | 4.38E-03 | 6 |  |
| lipopolysaccharide-mediated signaling pathway (GO:0031663) | 8.9 | 3.29E-05 | 1.57E-03 | 7 |  |
| negative regulation of execution phase of apoptosis (GO:1900118) | 8.9 | 4.55E-04 | 1.55E-02 | 5 |  |
| positive regulation of myoblast fusion (GO:1901741) | 8.9 | 1.73E-03 | 4.58E-02 | 4 |  |
| microglial cell activation (GO:0001774) | 8.61 | 1.43E-04 | 5.82E-03 | 6 |  |
| positive regulation of epidermal growth factor receptor signaling pathway (GO:0045742) | 8.61 | 1.43E-04 | 5.85E-03 | 6 |  |
| regulation of alpha-beta T cell proliferation (GO:0046640) | 8.34 | 1.66E-04 | 6.64E-03 | 6 |  |
| positive regulation of osteoclast differentiation (GO:0045672) | 8.24 | 6.20E-04 | 2.03E-02 | 5 |  |
| positive regulation of p38MAPK cascade (GO:1900745) | 8.24 | 6.20E-04 | 2.03E-02 | 5 |  |
| receptor signaling pathway via JAK-STAT (GO:0007259) | 8.19 | 5.23E-05 | 2.42E-03 | 7 |  |
| cellular zinc ion homeostasis (GO:0006882) | 8.09 | 1.93E-04 | 7.50E-03 | 6 |  |
| antigen processing and presentation of endogenous antigen (GO:0019883) | 7.94 | 7.18E-04 | 2.27E-02 | 5 |  |
| inflammatory response to antigenic stimulus (GO:0002437) | 7.94 | 7.18E-04 | 2.28E-02 | 5 |  |
| positive regulation of signaling receptor activity (GO:2000273) | 7.63 | 2.56E-04 | 9.56E-03 | 6 |  |
| T cell migration (GO:0072678) | 7.41 | 9.47E-04 | 2.84E-02 | 5 |  |
| positive regulation of immunoglobulin mediated immune response (GO:0002891) | 7.21 | 3.35E-04 | 1.20E-02 | 6 |  |
| positive regulation of cytokine biosynthetic process (GO:0042108) | 7.09 | 1.38E-06 | 8.29E-05 | 11 |  |
| cellular response to interferon-gamma (GO:0071346) | 7.05 | 1.04E-13 | 2.18E-11 | 26 |  |
| killing of cells of other organism (GO:0031640) | 6.97 | 1.83E-07 | 1.36E-05 | 13 |  |
| regulation of vascular endothelial growth factor production (GO:0010574) | 6.95 | 1.23E-03 | 3.50E-02 | 5 |  |
| protein kinase B signaling (GO:0043491) | 6.84 | 4.31E-04 | 1.49E-02 | 6 |  |
| cellular response to interleukin-1 (GO:0071347) | 6.82 | 7.05E-14 | 1.56E-11 | 27 |  |
| positive regulation of interferon-gamma production (GO:0032729) | 6.74 | 6.26E-06 | 3.40E-04 | 10 |  |
| regulation of vascular endothelial growth factor receptor signaling pathway (GO:0030947) | 6.74 | 1.39E-03 | 3.86E-02 | 5 |  |
| positive regulation of CD4-positive, alpha-beta T cell activation (GO:2000516) | 6.54 | 1.56E-03 | 4.20E-02 | 5 |  |
| pyridine-containing compound metabolic process (GO:0072524) | 6.51 | 5.47E-04 | 1.82E-02 | 6 |  |
| cellular response to interleukin-6 (GO:0071354) | 6.35 | 1.75E-03 | 4.62E-02 | 5 |  |
| ovulation cycle process (GO:0022602) | 6.21 | 6.87E-04 | 2.22E-02 | 6 |  |
| positive regulation of smooth muscle cell proliferation (GO:0048661) | 6.07 | 2.02E-06 | 1.19E-04 | 12 |  |
| negative regulation of extrinsic apoptotic signaling pathway (GO:2001237) | 5.99 | 3.30E-07 | 2.30E-05 | 14 |  |
| response to copper ion (GO:0046688) | 5.93 | 8.53E-04 | 2.59E-02 | 6 |  |
| positive regulation of T cell proliferation (GO:0042102) | 5.9 | 1.01E-06 | 6.36E-05 | 13 |  |
| positive regulation of T cell mediated immunity (GO:0002711) | 5.88 | 3.35E-04 | 1.20E-02 | 7 |  |
| regulation of nitric-oxide synthase activity (GO:0050999) | 5.8 | 9.46E-04 | 2.86E-02 | 6 |  |
| positive regulation of leukocyte mediated cytotoxicity (GO:0001912) | 5.66 | 4.11E-04 | 1.43E-02 | 7 |  |
| cellular response to mechanical stimulus (GO:0071260) | 5.56 | 2.85E-05 | 1.37E-03 | 10 |  |
| positive regulation of blood vessel endothelial cell migration (GO:0043536) | 5.56 | 4.54E-04 | 1.55E-02 | 7 |  |
| regulation of extrinsic apoptotic signaling pathway in absence of ligand (GO:2001239) | 5.56 | 1.16E-03 | 3.35E-02 | 6 |  |
| substrate adhesion-dependent cell spreading (GO:0034446) | 5.56 | 1.16E-03 | 3.35E-02 | 6 |  |
| positive regulation of cytokine secretion (GO:0050715) | 5.48 | 2.38E-08 | 2.05E-06 | 18 |  |
| positive regulation of oxidoreductase activity (GO:0051353) | 5.46 | 5.00E-04 | 1.69E-02 | 7 |  |
| regulation of interleukin-12 production (GO:0032655) | 5.46 | 5.00E-04 | 1.69E-02 | 7 |  |
| negative regulation of blood coagulation (GO:0030195) | 5.34 | 1.40E-03 | 3.89E-02 | 6 |  |
| regulation of bone remodeling (GO:0046850) | 5.34 | 1.40E-03 | 3.90E-02 | 6 |  |
| cytokine secretion (GO:0050663) | 5.23 | 1.54E-03 | 4.15E-02 | 6 |  |
| regulation of insulin receptor signaling pathway (GO:0046626) | 5.1 | 7.24E-04 | 2.28E-02 | 7 |  |
| negative regulation of cell-substrate adhesion (GO:0010812) | 5.02 | 7.91E-04 | 2.44E-02 | 7 |  |
| acute inflammatory response (GO:0002526) | 4.87 | 4.06E-04 | 1.42E-02 | 8 |  |
| positive regulation of T cell differentiation (GO:0045582) | 4.82 | 1.92E-04 | 7.49E-03 | 9 |  |
| positive regulation of protein kinase B signaling (GO:0051897) | 4.71 | 1.95E-07 | 1.43E-05 | 18 |  |
| positive regulation of ERK1 and ERK2 cascade (GO:0070374) | 4.68 | 9.46E-09 | 8.45E-07 | 22 |  |
| positive regulation of response to wounding (GO:1903036) | 4.45 | 1.52E-03 | 4.14E-02 | 7 |  |
| regulation of epithelial cell differentiation (GO:0030856) | 4.35 | 1.01E-05 | 5.30E-04 | 14 |  |
| viral entry into host cell (GO:0046718) | 4.26 | 4.49E-04 | 1.53E-02 | 9 |  |
| negative regulation of cysteine-type endopeptidase activity involved in apoptotic process (GO:0043154) | 4.19 | 1.02E-03 | 3.04E-02 | 8 |  |
| positive regulation of reactive oxygen species metabolic process (GO:2000379) | 4.04 | 6.36E-04 | 2.07E-02 | 9 |  |
| positive regulation of phosphatidylinositol 3-kinase signaling (GO:0014068) | 4.04 | 1.26E-03 | 3.57E-02 | 8 |  |
| tissue remodeling (GO:0048771) | 4 | 6.80E-04 | 2.21E-02 | 9 |  |
| positive regulation of angiogenesis (GO:0045766) | 3.95 | 1.46E-05 | 7.45E-04 | 15 |  |
| tumor necrosis factor-mediated signaling pathway (GO:0033209) | 3.74 | 5.74E-04 | 1.90E-02 | 10 |  |
| platelet activation (GO:0030168) | 3.57 | 4.43E-04 | 1.52E-02 | 11 |  |
| regulation of endothelial cell proliferation (GO:0001936) | 3.5 | 9.16E-04 | 2.78E-02 | 10 |  |
| pattern recognition receptor signaling pathway (GO:0002221) | 3.48 | 9.69E-04 | 2.89E-02 | 10 |  |
| negative regulation of secretion by cell (GO:1903531) | 3.32 | 9.31E-05 | 4.05E-03 | 15 |  |
| positive regulation of cell growth (GO:0030307) | 3.16 | 6.93E-04 | 2.22E-02 | 12 |  |
| placenta development (GO:0001890) | 3.14 | 1.21E-03 | 3.45E-02 | 11 |  |
| response to virus (GO:0009615) | 3.1 | 1.69E-05 | 8.52E-04 | 20 |  |
| negative regulation of cell activation (GO:0050866) | 3.1 | 3.08E-04 | 1.12E-02 | 14 |  |
| positive regulation of GTPase activity (GO:0043547) | 3.05 | 4.79E-07 | 3.22E-05 | 28 |  |
| T cell activation (GO:0042110) | 3.04 | 1.43E-04 | 5.81E-03 | 16 |  |
| angiogenesis (GO:0001525) | 2.93 | 2.33E-05 | 1.14E-03 | 21 |  |
| MAPK cascade (GO:0000165) | 2.69 | 5.05E-05 | 2.34E-03 | 22 |  |
| regulation of I-kappaB kinase/NF-kappaB signaling (GO:0043122) | 2.63 | 1.40E-03 | 3.89E-02 | 14 |  |
| negative regulation of cell migration (GO:0030336) | 2.57 | 1.20E-03 | 3.45E-02 | 15 |  |
| positive regulation of MAP kinase activity (GO:0043406) | 2.56 | 1.25E-03 | 3.54E-02 | 15 |  |
| positive regulation of ion transport (GO:0043270) | 2.51 | 1.02E-03 | 3.04E-02 | 16 |  |
| cellular calcium ion homeostasis (GO:0006874) | 2.49 | 7.60E-05 | 3.37E-03 | 25 |  |
| divalent metal ion transport (GO:0070838) | 2.48 | 8.37E-04 | 2.57E-02 | 17 |  |
| activation of protein kinase activity (GO:0032147) | 2.42 | 1.18E-03 | 3.40E-02 | 18 |  |
| positive regulation of apoptotic process (GO:0043065) | 2.37 | 7.41E-06 | 4.00E-04 | 35 |  |
| neutrophil activation (GO:0042119) | 2.33 | 1.10E-04 | 4.63E-03 | 26 |  |
| positive regulation of neurogenesis (GO:0050769) | 2.3 | 1.78E-04 | 7.05E-03 | 25 |  |
| negative regulation of cell population proliferation (GO:0008285) | 2.27 | 1.30E-05 | 6.67E-04 | 35 |  |
| myeloid leukocyte mediated immunity (GO:0002444) | 2.25 | 2.46E-04 | 9.20E-03 | 26 |  |
| actin cytoskeleton organization (GO:0030036) | 2.24 | 3.50E-04 | 1.24E-02 | 25 |  |
| response to peptide (GO:1901652) | 2.23 | 4.98E-04 | 1.69E-02 | 24 |  |
| response to toxic substance (GO:0009636) | 2.22 | 2.71E-04 | 9.94E-03 | 26 |  |
| supramolecular fiber organization (GO:0097435) | 2.16 | 1.18E-03 | 3.40E-02 | 22 |  |
| myeloid cell activation involved in immune response (GO:0002275) | 2.15 | 5.18E-04 | 1.74E-02 | 25 |  |
| regulation of cell morphogenesis (GO:0022604) | 2.14 | 7.13E-04 | 2.27E-02 | 24 |  |
| negative regulation of immune system process (GO:0002683) | 2.14 | 1.28E-03 | 3.59E-02 | 22 |  |
| leukocyte degranulation (GO:0043299) | 2.12 | 7.90E-04 | 2.44E-02 | 24 |  |
| cell population proliferation (GO:0008283) | 2 | 1.42E-03 | 3.92E-02 | 25 |  |
| apoptotic process (GO:0006915) | 1.86 | 3.76E-04 | 1.32E-02 | 38 |  |
| G protein-coupled receptor signaling pathway (GO:0007186) | 1.62 | 1.50E-03 | 4.10E-02 | 48 |  |
| positive regulation of gene expression (GO:0010628) | 1.53 | 5.11E-04 | 1.72E-02 | 69 |  |
| Unclassified (UNCLASSIFIED) | 0.41 | 4.00E-09 | 3.79E-07 | 29 |  |
| mRNA metabolic process (GO:0016071) | 0.19 | 2.99E-04 | 1.09E-02 | 3 |  |
| DNA repair (GO:0006281) | 0.17 | 1.26E-03 | 3.56E-02 | 2 |  |
| RNA processing (GO:0006396) | 0.15 | 8.50E-06 | 4.52E-04 | 3 |  |
|  |  |  |  |  |  |
|  |  |  |  |  |  |
| **GO terms associated with upregulated genes in HkLp-Lp compared to U-Lp** | | | | | |
| **GO biological process complete** | **Client Text Box Input (fold Enrichment)** | **Client Text Box Input (raw P-value)** | **Client Text Box Input (FDR)** | **Client Text Box Input (94)** |  |
| response to virus (GO:0009615) | 7.000 | 7.01E-06 | 1.39E-02 | 9.000 |  |
| response to cytokine (GO:0034097) | 3.400 | 9.77E-06 | 1.73E-02 | 17.000 |  |
| modification of morphology or physiology of other organism (GO:0035821) | 8.450 | 2.44E-05 | 3.52E-02 | 7.000 |  |
| metal ion homeostasis (GO:0055065) | 4.240 | 2.88E-05 | 3.53E-02 | 12.000 |  |
| innate immune response (GO:0045087) | 3.790 | 4.11E-05 | 4.67E-02 | 13.000 |  |
| regulation of immune system process (GO:0002682) | 2.680 | 4.42E-05 | 4.69E-02 | 20.000 |  |
|  |  |  |  |  |  |
|  |  |  |  |  |  |
| **GO terms associated with upregulated genes in HkLp-Lp compared to U-Lp** | | | | | |
| **GO biological process complete** | **Client Text Box Input (fold Enrichment)** | **Client Text Box Input (raw P-value)** | **Client Text Box Input (FDR)** | **Client Text Box Input (217)** |  |
| neutrophil chemotaxis (GO:0030593) | 18.65 | 4.41E-15 | 6.37E-12 | 16 |  |
| leukocyte chemotaxis (GO:0030595) | 12.51 | 7.95E-15 | 9.72E-12 | 19 |  |
| chemokine-mediated signaling pathway (GO:0070098) | 16.93 | 7.64E-13 | 3.04E-10 | 14 |  |
| monocyte chemotaxis (GO:0002548) | 25.34 | 5.21E-12 | 1.62E-09 | 11 |  |
| cellular response to lipopolysaccharide (GO:0071222) | 8.98 | 7.19E-12 | 2.16E-09 | 18 |  |
| cellular response to interleukin-1 (GO:0071347) | 7.15 | 7.97E-08 | 1.22E-05 | 13 |  |
| cell adhesion (GO:0007155) | 3.05 | 1.42E-07 | 2.06E-05 | 29 |  |
| cellular response to tumor necrosis factor (GO:0071356) | 5.6 | 4.25E-07 | 5.63E-05 | 14 |  |
| Unclassified (UNCLASSIFIED) | 0.31 | 1.93E-06 | 2.24E-04 | 10 |  |
| positive regulation of angiogenesis (GO:0045766) | 6.3 | 2.59E-06 | 2.90E-04 | 11 |  |
| positive regulation of ERK1 and ERK2 cascade (GO:0070374) | 5.56 | 3.11E-06 | 3.41E-04 | 12 |  |
| eosinophil chemotaxis (GO:0048245) | 24.19 | 4.81E-06 | 5.03E-04 | 5 |  |
| positive regulation of tyrosine phosphorylation of STAT protein (GO:0042531) | 9.96 | 1.17E-05 | 1.13E-03 | 7 |  |
| extrinsic apoptotic signaling pathway (GO:0097191) | 7.98 | 1.26E-05 | 1.20E-03 | 8 |  |
| cellular response to interferon-gamma (GO:0071346) | 5.9 | 1.28E-05 | 1.22E-03 | 10 |  |
| myeloid leukocyte activation (GO:0002274) | 3.16 | 1.42E-05 | 1.33E-03 | 19 |  |
| positive regulation of adaptive immune response based on somatic recombination of immune receptors built from immunoglobulin superfamily domains (GO:0002824) | 7.59 | 1.77E-05 | 1.60E-03 | 8 |  |
| antimicrobial humoral immune response mediated by antimicrobial peptide (GO:0061844) | 7.44 | 2.01E-05 | 1.79E-03 | 8 |  |
| negative regulation of blood coagulation (GO:0030195) | 11.61 | 2.27E-05 | 1.95E-03 | 6 |  |
| positive regulation of neuroinflammatory response (GO:0150078) | 27.64 | 2.91E-05 | 2.41E-03 | 4 |  |
| killing of cells of other organism (GO:0031640) | 8.16 | 3.87E-05 | 3.05E-03 | 7 |  |
| angiogenesis (GO:0001525) | 3.94 | 4.04E-05 | 3.15E-03 | 13 |  |
| detoxification of copper ion (GO:0010273) | 24.19 | 4.54E-05 | 3.45E-03 | 4 |  |
| cell-cell signaling (GO:0007267) | 2.35 | 4.77E-05 | 3.59E-03 | 27 |  |
| positive regulation of interleukin-17 production (GO:0032740) | 22.77 | 5.56E-05 | 4.07E-03 | 4 |  |
| regulation of microglial cell activation (GO:1903978) | 21.5 | 6.74E-05 | 4.81E-03 | 4 |  |
| positive regulation of leukocyte chemotaxis (GO:0002690) | 7.44 | 6.69E-05 | 4.82E-03 | 7 |  |
| retinal ganglion cell axon guidance (GO:0031290) | 20.37 | 8.10E-05 | 5.57E-03 | 4 |  |
| positive regulation of interferon-gamma production (GO:0032729) | 8.8 | 9.52E-05 | 6.47E-03 | 6 |  |
| positive regulation of protein kinase B signaling (GO:0051897) | 5.12 | 9.82E-05 | 6.61E-03 | 9 |  |
| positive regulation of T cell proliferation (GO:0042102) | 6.91 | 1.04E-04 | 6.90E-03 | 7 |  |
| positive regulation of GTPase activity (GO:0043547) | 3.32 | 1.21E-04 | 7.74E-03 | 14 |  |
| hyaluronan biosynthetic process (GO:0030213) | 41.47 | 1.20E-04 | 7.77E-03 | 3 |  |
| ectopic germ cell programmed cell death (GO:0035234) | 41.47 | 1.20E-04 | 7.80E-03 | 3 |  |
| positive regulation of cytokine biosynthetic process (GO:0042108) | 8.41 | 1.20E-04 | 7.80E-03 | 6 |  |
| collagen catabolic process (GO:0030574) | 11.25 | 1.28E-04 | 8.08E-03 | 5 |  |
| regulation of bone resorption (GO:0045124) | 11.25 | 1.28E-04 | 8.11E-03 | 5 |  |
| negative regulation of extrinsic apoptotic signaling pathway (GO:2001237) | 6.51 | 1.47E-04 | 9.06E-03 | 7 |  |
| regulation of cell growth (GO:0001558) | 3.23 | 1.62E-04 | 9.84E-03 | 14 |  |
| cellular response to zinc ion (GO:0071294) | 16.13 | 1.80E-04 | 1.06E-02 | 4 |  |
| RNA processing (GO:0006396) | < 0,01 | 1.98E-04 | 1.15E-02 | 0 |  |
| positive regulation of neurogenesis (GO:0050769) | 3 | 2.00E-04 | 1.16E-02 | 15 |  |
| wound healing (GO:0042060) | 2.99 | 2.09E-04 | 1.20E-02 | 15 |  |
| positive regulation of vascular endothelial growth factor production (GO:0010575) | 13.82 | 3.06E-04 | 1.67E-02 | 4 |  |
| negative regulation of growth (GO:0045926) | 3.93 | 3.20E-04 | 1.72E-02 | 10 |  |
| regulation of epithelial to mesenchymal transition (GO:0010717) | 6.91 | 3.26E-04 | 1.74E-02 | 6 |  |
| negative regulation of collagen biosynthetic process (GO:0032966) | 26.39 | 3.54E-04 | 1.84E-02 | 3 |  |
| T cell cytokine production (GO:0002369) | 26.39 | 3.54E-04 | 1.84E-02 | 3 |  |
| response to vitamin D (GO:0033280) | 13.35 | 3.45E-04 | 1.84E-02 | 4 |  |
| negative regulation of cell population proliferation (GO:0008285) | 2.54 | 3.52E-04 | 1.85E-02 | 18 |  |
| secretion by cell (GO:0032940) | 2.22 | 3.75E-04 | 1.93E-02 | 23 |  |
| cellular response to copper ion (GO:0071280) | 12.9 | 3.88E-04 | 1.98E-02 | 4 |  |
| positive regulation of myeloid leukocyte differentiation (GO:0002763) | 8.34 | 4.64E-04 | 2.33E-02 | 5 |  |
| cellular zinc ion homeostasis (GO:0006882) | 11.73 | 5.40E-04 | 2.69E-02 | 4 |  |
| viral entry into host cell (GO:0046718) | 6.18 | 5.73E-04 | 2.82E-02 | 6 |  |
| intracellular protein transport (GO:0006886) | 0.1 | 5.68E-04 | 2.82E-02 | 1 |  |
| positive regulation of protein kinase activity (GO:0045860) | 2.71 | 5.79E-04 | 2.84E-02 | 15 |  |
| positive regulation of tissue remodeling (GO:0034105) | 11.38 | 5.99E-04 | 2.88E-02 | 4 |  |
| positive regulation of aldosterone secretion (GO:2000860) | 96.76 | 6.17E-04 | 2.94E-02 | 2 |  |
| reproductive structure development (GO:0048608) | 2.95 | 6.36E-04 | 3.02E-02 | 13 |  |
| positive regulation of leukocyte mediated immunity (GO:0002705) | 5.02 | 6.59E-04 | 3.08E-02 | 7 |  |
| regulation of cell killing (GO:0031341) | 5.86 | 7.42E-04 | 3.40E-02 | 6 |  |
| extracellular matrix disassembly (GO:0022617) | 7.44 | 7.55E-04 | 3.44E-02 | 5 |  |
| positive regulation of protein secretion (GO:0050714) | 3.46 | 8.44E-04 | 3.77E-02 | 10 |  |
| regulation of cell size (GO:0008361) | 4.18 | 8.63E-04 | 3.82E-02 | 8 |  |
| cellular response to cadmium ion (GO:0071276) | 10.18 | 8.79E-04 | 3.88E-02 | 4 |  |
| positive regulation of vascular endothelial growth factor receptor signaling pathway (GO:0030949) | 18.14 | 9.06E-04 | 3.98E-02 | 3 |  |
| T cell activation (GO:0042110) | 3.72 | 9.27E-04 | 4.06E-02 | 9 |  |
| positive regulation of cytosolic calcium ion concentration involved in phospholipase C-activating G protein-coupled signaling pathway (GO:0051482) | 9.92 | 9.62E-04 | 4.19E-02 | 4 |  |
| regulation of neuron projection development (GO:0010975) | 2.68 | 9.79E-04 | 4.25E-02 | 14 |  |
| regulation of platelet-derived growth factor production (GO:0090361) | 64.5 | 1.02E-03 | 4.41E-02 | 2 |  |
| fever generation (GO:0001660) | 64.5 | 1.02E-03 | 4.42E-02 | 2 |  |
| regulation of hormone metabolic process (GO:0032350) | 9.68 | 1.05E-03 | 4.52E-02 | 4 |  |
| regulation of interferon-gamma biosynthetic process (GO:0045072) | 17.07 | 1.06E-03 | 4.55E-02 | 3 |  |
| positive regulation of epithelial cell proliferation (GO:0050679) | 4.03 | 1.09E-03 | 4.65E-02 | 8 |  |
| pyridine-containing compound metabolic process (GO:0072524) | 9.44 | 1.14E-03 | 4.83E-02 | 4 |  |
| regulation of keratinocyte differentiation (GO:0045616) | 9.44 | 1.14E-03 | 4.85E-02 | 4 |  |
