# Supplementary material for: The commensal bacterium Lactiplantibacillus plantarum imprints innate memory-like responses in mononuclear phagocytes
Source: Gut Microbes. 2021 Jul 5;13(1):1939598. doi: 10.1080/19490976.2021.1939598 (PMC8259724; doi:10.1080/19490976.2021.1939598)
Supplement: Supplemental Material [file KGMI_A_1939598_SM6562.zip › supplementary/Pellon_Anguita_Supplementary_Revised.docx]

SUPPLEMENTARY MATERIALS

**The commensal bacterium *Lactiplantibacillus plantarum* imprints innate memory-like responses in mononuclear phagocytes**

Aize Pellon^1,&,#^, Diego Barriales^1,#^, Ainize Peña-Cearra^1,2^, Janire Castelo-Careaga^1^, Ainhoa Palacios^1^, Nerea Lopez^1^, Estibaliz Atondo^1^, Miguel Angel Pascual-Itoiz^1^, Itziar Martín-Ruiz^1^, Leticia Sampedro^1^, Monika Gonzalez-Lopez^3^, Laura Bárcena^3^, Teresa Martín-Mateos^4^, Jose María Landete^5^, Rafael Prados-Rosales^1,&^, Laura Plaza-Vinuesa^6^, Rosario Muñoz^6^, Blanca de las Rivas^6^, Juan Miguel Rodríguez^7^, Edurne Berra^4^, Ana M. Aransay^3,8^, Leticia Abecia^1,2^, Jose Luis Lavín^9,&^, Hector Rodríguez^1,*^ and Juan Anguita^1,10,*^

^1^ Inflammation and Macrophage Plasticity Laboratory, CIC bioGUNE-BRTA (Basque Research and Technology Alliance), Derio 48160, Spain

^2^ Faculty of Medicine and Nursing, Universidad del Pais Vasco (UPV/EHU), Leioa 48940, Spain.

^3^ Genomic Analysis platform, CIC bioGUNE-BRTA, Derio, Spain 48160

^4^ Physiopathology of the hypoxia-signaling pathway laboratory, CIC bioGUNE-BRTA, Derio, Spain 48160

^5^ Departamento de Tecnología de Alimentos, Instituto Nacional de Investigación y Tecnología Agraria y Alimentaria (INIA), Madrid 28040, Spain.

^6^ Laboratorio de Biotecnología Bacteriana, Instituto de Ciencia y Tecnología de Alimentos y Nutrición (ICTAN-CSIC), Madrid 28006, Spain

^7^ Department of Nutrition and Food Science, Universidad Complutense de Madrid, Madrid 28040, Spain.

^8^ CIBERehd, ISCIII, Madrid, Spain.

^9^ Bioinformatics Unit, CIC bioGUNE-BRTA, Derio 48160, Spain.

^9^ Ikerbasque, Basque Foundation for Science. Bilbao 48013, Bizkaia, Spain.

**Supplementary figure 1. Experimental setups and bacterial intracellular survival in murine cell models. (**A) Experimental setup of the antibiotic protection assays used to evaluate bacterial intracellular survival. Bacteria were added to monocyte/macrophage cultures, and kept at 4 °C for 30 min. The plates were then incubated at 37 °C, 5% CO_2_ for 45 min, washed to eliminate extracellular bacteria and added antibiotics. After 1, 4 or 24 h of antibiotic treatment, samples from the supernatant and cell lysates were obtained and incubated on agar plates to quantify bacterial numbers (colony forming units; cfu). Intracellular survival of the different bacterial strains was evaluated using the murine macrophage cell line RAW264.6 at a multiplicity of infection (m.o.i.) of 10 (B) or 1 (C), or mBMMs at an m.o.i. = 1 (D). Data are shown as mean ± s.e.m., n ≥ 4. (E) Experimental setup for the evaluation of the bacterial ability to extrude from immune cells. One or 4 h after the addition of antibiotics, the cells were extensively washed, and further incubated in antibiotic-free medium. After 24 h, samples from the supernatants or cell lysates were plated on agar to quantify bacteria (cfu). ***; p < 0.001, two-way ANOVA.

**Supplementary figure 2. Induction of innate memory by other *L. plantarum* strains and *L. casei*.** TNF induction by the *L. plantarum* strains MP31 (A) and MP33 (B) in mBMMs. The cells were either pre-stimulated with the bacteria (Lp MP31-Lp MP31; Lp MP33-Lp MP33) or left unstimulated during the priming process (U-Lp MP31; U-Lp MP33). (C) Intracellular survival of *L. plantarum* in mBMMs primed with MRS medium (MRS-Lp), conditioned medium from an overnight culture of *L. plantarum* (MRS+Lp-Lp), *L. plantarum* (Lp-Lp) or left unstimulated (U-Lp). (D) Intracellular survival of *L. casei* in mBMMs previously stimulated with the bacteria (Lc-Lc) or left unstimulated during the priming process (U-Lp). (E) TNF quantification in the stimulation supernatants of prestimulated and non prestimulated mBMMs with *L. casei*. The data are presented as means ± s.e.m., n ≥ 3. *; p < 0.05, ***; p < 0.001, two-way ANOVA (C) and Student´s t test (A,B,D).

**Supplementary figure 3. *L. plantarum* priming induces changes in the metabolic profile of murine macrophages.** Oxygen consumption rate (OCR; A) and extracellular acidification rate (ECAR; B) of mBMMs prestimulated with either live (Lp-Lp) or heat-killed (HKLp-Lp) *L. plantarum* WCSF1 and restimulated with live bacteria, compared to unprimed controls (U-Lp). Data are shown for a representative experiment out of two independently performed. (C) Phenogram showing OCR/ECAR ratios of the three experimental groups. Lactate concentration in the supernatants of unprimed or pre-stimulated human monocytes (D) and mBMMs (E). The data represent the average ± s.e.m., n ≥ 5.
